# Meningococcal Antigen Typing System Development and Application to the Evaluation of Effectiveness of Meningococcal B Vaccine and Possible Use for Other Purposes

**DOI:** 10.1155/2015/353461

**Published:** 2015-08-17

**Authors:** Alexander Domnich, Roberto Gasparini, Daniela Amicizia, Giuseppe Boccadifuoco, Marzia Monica Giuliani, Donatella Panatto

**Affiliations:** ^1^Department of Health Sciences, University of Genoa, Via Pastore 1, 16132 Genoa, Italy; ^2^Novartis Vaccines, Via Fiorentina 1, 53100 Siena, Italy

## Abstract

Development of the 4-component meningococcal serogroup B vaccine (4CMenB) has required new assays for the reliable evaluation of the expression and cross-reactivity of those specific antigen variants that are predicted to be targeted by bactericidal antibodies elicited by the vaccine in different isolates. Existing laboratory techniques, such as multilocus sequence typing, are poorly suited to this purpose, since they do not provide information on the contribution of single vaccine components and therefore cannot be applied to estimate the potential coverage of the multicomponent vaccine. The hSBA, the only correlate of protection against invasive meningococcal disease accepted thus far, cannot conveniently be used to test large number of strains. To overcome these issues, the meningococcal antigen typing system (MATS) has been specifically developed in order to predict 4CMenB coverage of individual meningococcus serogroup B strains. To date, MATS has proved advantageous for several reasons, including its ability to assess both qualitative and quantitative aspects of surface antigens of single strains in a highly reproducible, rapid, and resource-saving manner, while its shortcomings include a possible underestimation of 4CMenB coverage and the use of pooled sera to calculate the positive bactericidal threshold. This paper provides an overview of MATS development and its field application.

## 1. Introduction

**Figure 1:**
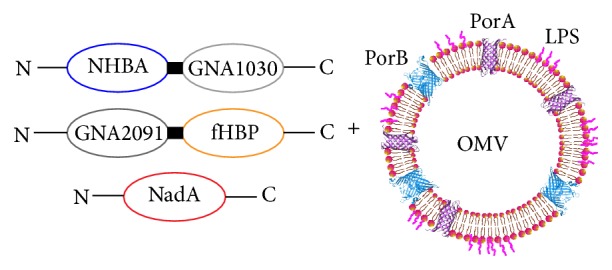
4CMenB vaccine composition. Antigens NHBA and fHbp are fused with two accessory proteins, GNA1030 and GNA2091, respectively. Adapted with permission from [15].

*Neisseria meningitidis* is a major causative agent of invasive bacterial diseases that affect mostly children between 3 and 12 months of age, followed by adolescents. Of thirteen known serogroups of* N. meningitidis*, only six (A, B, C, W-135, X, and Y) cause invasive disease [1, 2]. Active immunization is the most effective way to prevent invasive meningococcal disease; vaccines against serogroups A, C, W-135, and Y and a recently approved universal vaccine against serogroup B (MenB) are available [3]. This latter, a 4-component meningococcal serogroup B vaccine (4CMenB, commercially available as Bexsero), is the first vaccine to be developed by means of reverse vaccinology [4, 5]. 4CMenB consists of three recombinant proteins, namely, factor H binding protein (fHbp), Neisserial heparin-binding antigen (NHBA), and* Neisseria* adhesin A (NadA), combined with OMV from MenB strain NZ98/254, which contains porin A (PorA) serosubtype P1.4 (see Figure 1) [6].

Evaluating the protective efficacy of a vaccine without measuring clinical outcomes is of great practical importance [7, 8]. This is particularly true for vaccines against* N. meningitidis*, the incidence of which is relatively low [9, 10]. A correlate or surrogate of protection can be defined as an immunological measurement that correlates statistically with the level of a trial endpoint used to measure vaccine efficacy [11]. The serum bactericidal assay with human complement (hSBA) is a universally accepted correlate of protection against meningococcal disease that quantifies the complement-mediated killing of bacteria by functional antibodies in sera from vaccinees [12]; in general, an hSBA titer ≥ 1 : 4 is considered to be a correlate of protection [12, 13].

Despite its strengths, hSBA has some shortcomings. First, hSBA is a labor-intensive technique and testing a large number of single circulating strains would produce logistical difficulties. Second, it requires collecting considerable amounts of sera from immunized individuals, which would be ethically debatable, especially in pediatric studies. Third, the standardization of hSBA across numerous strains and complement sources is also burdensome [5, 14, 15]. Fourth, while hSBA is able to assess the effectiveness of a vaccine by measuring bactericidal antibody titers, it does not provide information on the contribution of each vaccine component [14]. Indeed, the surface-exposed proteins fHbp, NHBA, and NadA of MenB display considerable sequence variation and expression, as well as different degrees of cross-reactivity among variants of a protein antigen to the antibodies induced by the vaccine [16–20]. We therefore need new assays that can reliably assess the expression of those specific antigen variants that are predicted to be targeted by bactericidal antibodies elicited by the vaccine on different bacterial isolates.

Today, the most widely used approach for characterizing single meningococci is multilocus sequence typing (MLST), which defines strains from the sequences of seven housekeeping genes, including* arcC* (carbamate kinase),* aroE* (shikimate dehydrogenase),* glpF* (glycerol kinase),* gmk* (guanylate kinase), pta (phosphate acetyltransferase),* tpi* (triosephosphate isomerase), and* yqiL* (acetyl coenzyme A acetyltransferase) [21, 22]. It should, however, be noted that the classification of strains based on MLST does not give direct indication on the 4CMenB antigenic repertoire. A study by Bambini et al. [18] demonstrated that each MLST clonal complex has an almost specific antigen variant repertoire resulting in a weak correlation between MLST and antigenic variability. It has been confirmed that the clonal complex alone generally has no discriminatory power to predict which strain will be killed on hSBA. These considerations make MLST only partially suitable for determining the phenotype profile, predicting vaccine antigen diversity, and, thus, assessing potential strain coverage.

Another potentially useful method to predict strain coverage is flow cytometry, which uses arrays of mono- and polyclonal antibodies and enables a considerable number of strains to be analyzed; currently, however, the method is implemented in few laboratories and may have standardization difficulties and, by using monoclonal antibodies only, it gives indication on the amount of antigens on the surface but not on their sequence diversity [5, 23].

To overcome the aforementioned limitations, a novel approach, termed the meningococcal antigen typing system (MATS), has been developed [14], its main aim being to predict the coverage of individual MenB strains provided by vaccination with 4CMenB by measuring the amount of antigen and its cross-reactivity. At the same time, since most capsular strains of* N. meningitidis* may express the same protein antigens [24, 25], the application of MATS could be extended to other serogroups. Moreover, this technique could be potentially adapted to other bacterial pathogens [26]. For these reasons recent advances and applications of MATS in the field of epidemiologic surveillance of bacteria should now be reviewed.

## 2. MATS Development and Interpretation

### 2.1. MATS as a Qualitative and Quantitative Assay

MATS was designed as a rapid and robust binding assay able to predict the susceptibility of individual MenB strains to be killed by bactericidal antibodies elicited by 4CMenB; this method enables both qualitative (level of sequence relatedness) and quantitative (level of expression) evaluation of the antigens expressed on the surface of single strains [14, 15]. Both quantitative and qualitative aspects are highly important and should be assessed for the reasons described below.

The density and spatial orientation of an antigen on the bacterial surface are critical in the process of classical pathway of complement activation, which is initiated when a sufficient density of antigen-antibody complexes allows proximate fragment crystallizable (Fc) regions of the antibody to bind C1q. An increase in surface antigen density results in a reduced distance between bound antibodies, thus leading to a higher probability of engagement and activation of the complement system [27, 28]. On the other hand, the level of surface antigen expression is not the only factor that influences killing; the quality of the fit antigen-antibody is also crucial [29].

Basically, MATS is a modified sandwich enzyme-linked immunosorbent assay (ELISA) that quantifies expression and the level of matching with the corresponding antigen in the vaccine (fHbp, NHBA, and NadA) on bacterial lysates. Moreover, the PorA serosubtype is identified by means of the traditional PCR genotypic approach by assessing its variable region 2, and an individual strain matching for PorA (PorA 1.4) is considered to be covered by 4CMenB. This subsequently enables antigenic cross-reactivity with the main corresponding 4CMenB components to be measured [14, 15].

**Figure 2:**
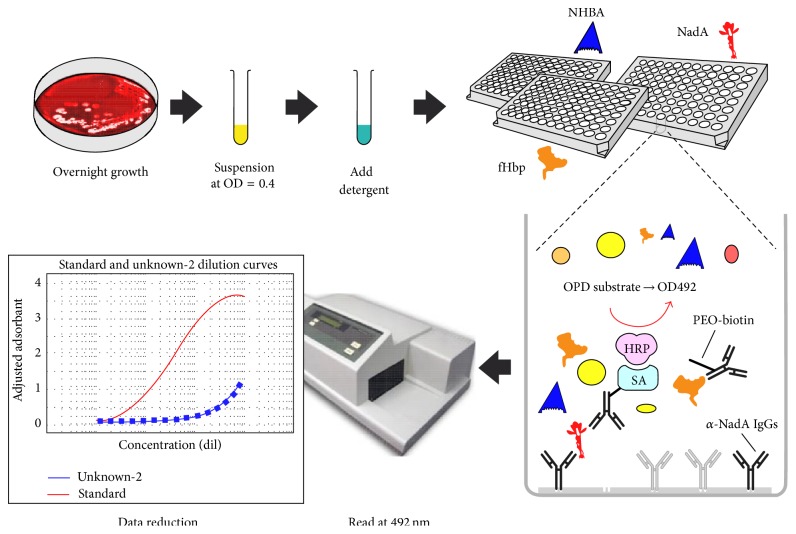
Schematic representation of the MATS ELISA method [15]. Bacteria from overnight cultures on agar chocolate plates are suspended in Mueller-Hinton broth and lysed with a detergent (Empigen BB 5%) added to a final volume of 1/11 and inactivated for 1 hour at 45°C in a water bath; bacterial lysates are added to three different ELISA microwell plates coated with polyclonal rabbit antibodies raised against the single vaccine components fHbp, NHBA, and NadA; the antigens are captured from the suspension to the plate. Plates are then incubated for 1 hour at 37°C with biotinylated rabbit polyclonal antibodies against each of the antigens, washed, incubated with streptavidin-HRP, and developed with the OPD substrate. Relative potency is calculated by interpolating the regression curve of the unknown sample versus that of a reference strain added to each plate. Adapted with permission from [15].

Methodologically, the MATS ELISA procedure comprises several steps, which can be schematized as follows (see Figure 2). At the first step, cultures are grown overnight on chocolate agar plates at 37°C, 95% relative humidity, and CO_2_ concentration of 5%. Subsequently, bacteria are suspended in Mueller-Hinton broth to achieve an optical density (OD) at 600 nm of 0.4 and then lysed with the detergent Empigen BB 5% added to a final volume of 1 : 11 (0.45%) and inactivated at 45°C for 1 hour in a water bath. Twofold serial dilutions of the bacterial lysates are incubated in duplicate in three different ELISA microwell plates coated with rabbit polyclonal antibodies against fHbp, NHBA, and NadA, respectively. The plates are incubated for 1 hour at 37°C and washed with a solution of phosphate buffered saline (PBS1x) plus 0.05% Tween. The amount of antigen bound to the antibodies is detected by incubating for 1 hour at 37°C with purified rabbit immunoglobulin G (IgG) raised against each of the three recombinant proteins, labeled with biotin. The plates are then washed and incubated for 30 min at 37°C with streptavidin-horseradish peroxidase and for 20 min at room temperature with the* ortho*-phenylene diamine substrate; the reaction is stopped by adding 50 *μ*L of sulfuric acid solution (4 N). Immediately afterwards, the plates are read at 492 nm by means of an ELISA reader [14, 15].

The MATS ELISA readout expresses the relative potency (RP) for fHbp, NHBA, and NadA of single strains; RPs of tested strains are calculated by means of a variance-weighted regression method by comparing the serial dilution curves of tested strains with those of the appropriate reference strains for each of the three antigens. The reference strains are as follows: H44/76 for fHbp, NGH38 for NHBA, and 5/99 for NadA; the RPs are calculated by assigning the arbitrary value of 1 (or 100%) to each reference strain. Subsequently, in order to determine cut-off values of RPs that would predict susceptibility on hSBA, MATS RPs have been related to hSBA of pooled serum from 13-month-old children immunized with 4CMenB at 2, 4, 6, and 12 months of age. The positive bactericidal threshold (PBT) has been defined as the MATS RP point estimate, above which the majority of strains are killed in hSBA. These RP values are 0.021 (2.1%) for fHbp, 0.294 (29.4%) for NHBA, and 0.009 (0.9%) for NadA [14]. In sum, the MATS phenotype is defined as follows: each of the four antigens can be either positive (when RP > PBT or PorA is P1.4) or negative (when RP ≤ PBT and PorA is not P1.4) so that the number of possible MATS phenotypes is 2^4^ = 16 [14, 30].

### 2.2. Impact of Antigen Sequence Variation on the Relative Potency Values (Qualitative Aspects of MATS)

As described above, the level of sequence relatedness of an antigen in a given MenB strain to the corresponding antigen included in the vaccine is a determining factor. The main 4CMenB antigens, fHbp, NHBA, and NadA, have a substantial level of sequence variability [16–18]. For instance, fHbp has been divided into three variants, namely, 1, 2, and 3, each of which may be further classified into subvariants. Sequence conservation within variants is high (91.6–100%), while that between variants is approximately 63% [17, 18]. Other fHbp classifications based on two subfamilies [31] and nine modular groups [20] have also been described. Similarly, several peptides of NHBA and at least five NadA variants have been established [16]. 4CMenB recombinant proteins include fHbp subvariant 1.1, NHBA subvariant 2, and NadA variant 3 [24]. MATS RPs of single antigens in MenB strains are highly influenced by such complex genetic variations. In a panel of 124 MenB isolates, selected to represent a broad range of MLST and PorA types from different geographic areas, the highest RPs of fHbp (46–140%) have been found among strains classified within fHbp subvariant 1.1. Strains expressing other subvariants of the fHbp variant 1 have shown significantly lower RPs of 1.6–38%, while those expressing fHbp variants 2 and 3 have displayed RPs below the lower limit of quantitation. Although all tested strains harbor the* nhba* gene, only 70% of them have RPs above the lower limit of quantitation and a 6.5-fold range in RPs has been observed (from 20% to 130%). The* nadA* gene has been found in only one-third of isolates and less than 20% showed RPs above the lower limit of quantitation, with a more than 1000-fold range in RP values [14].

Analogous results have been reported from Canada, where all isolates expressing fHbp subvariant 1.1 showed RPs > PBT, while the proportions of strains predicted to be covered by 4CMenB were 33%, 85%, and 95% for those expressing the more distant subvariants 1.13, 1.15, and 1.4, respectively. None of the fHbp variant 2 or 3 strains had RPs above the PBT for fHbp and would require expression of a different vaccine antigen (i.e., PorA, NHBA, and NadA) in order to be covered [32]. Among European MenB isolates, strains with different subvariants of fHbp-1 have generally shown RPs > PBT, while almost all strains with fHbp-2 or fHbp-3 have had RPs close to 0% or, in any case, below the PBT. A high variation in RPs for different variants of NHBA has also been documented in three studies [5, 32, 33].

### 2.3. Association between MATS and hSBA

To investigate the relationship between MATS RPs and bactericidal titers, 57 strains from the 124 MenB panel were tested by means of hSBA using pooled infant serum from 13-month-old children immunized with 4CMenB at 2, 4, 6, and 12 months of age. To assess the contribution of each antigen to the bactericidal activity, a subset of 5 strains for fHbp, 11 for NHBA, and 7 for NadA that were mismatched to the vaccine for PorA and had only one vaccine antigen RP above the MATS lower limit of quantitation was selected. In this subset of strains the correlations between hSBA titers and relative potencies were statistically significant. Spearman's correlation coefficients were as follows: *ρ* = 0.97 (*P* = 0.005) for fHbp; *ρ* = 0.75 (*P* = 0.008) for NHBA; *ρ* = 0.81 (*P* = 0.027) for NadA. Eighty-nine percent (39/44) of strains with RPs above the PBT for at least one antigen have been seen to be killed on hSBA (titer of ≥ 1 : 8 or 4-fold rise) with pooled sera from 13-month-old children immunized with four 4CMenB doses. When considering RPs above the PBT for the single antigens, the highest positive predictive value was for fHbp (100%, 7/7), followed by NadA (83%, 5/6) and NHBA (82%, 9/11). The negative predictive value (proportion of strains with all four RPs ≤ PBT that are not killed on hSBA) was also high (77%, *n* = 13) [14]. Similar findings have been observed among adults who received three 4CMenB doses: within the 124-strain panel, 83 out of 91 (91%) strains showing RPs above the PBT for one or more antigens were killed on hSBA with pooled sera. Positive predictive values increased as the number of antigens with RP > PBT increased (1 antigen: 85%, *n* = 41; 2 antigens: 94%, *n* = 34; 3 antigens: 100%, *n* = 16) [14].

The use of pooled immune sera, however, may not accurately predict how each of the sera that compose the pool would react against a tested strain. Indeed, it is possible that a few subjects with unusually high or low hSBA titers may impact on the overall pooled response or that the potential synergy between antibodies from different subjects might generate a bactericidal response which would not be achieved individually. However, a recent study [34] aiming at investigating the relationship between pooled and individual sera has shown that individual responses to 4CMenB are homogeneously distributed and that pooled hSBA titers reflect the arithmetic mean of the individual titers with good approximation.

### 2.4. Reliability and Reproducibility of MATS

Interlaboratory standardization of MATS has been also achieved by using 17 shared MenB strains [26]. The log-transformed RPs obtained from five laboratories showed excellent robustness at different temperatures of sample inactivation (37°C and 45°C), with Lin's coefficient of accuracy being 0.999 (95% CI: 0.997–1) and Pearson's correlation coefficient being 0.993 (95% CI: 0.988–0.995). Log-transformed RPs from seven laboratories displayed almost perfect accuracy and concordance, with the corresponding coefficients exceeding 0.99. Within-laboratory variations in RPs for individual antigens differed slightly, being highest for NadA and lowest for fHbp; this variance, however, was not affected by systematic bias. Similarly, between-laboratory coefficients of variation were 7.85% for fHbp, 12.60% for NHBA, and 16.51% for NadA. Moreover, in this study empirical estimates of 95% CI of PBTs (Table 1) for each individual antigen were calculated as 10^lg(PBT)±1.96•*σ*^. The authors proposed using 95% confidence limits to predict 4CMenB coverage, as these would account for between- and within-laboratory variances. Laboratory qualification criteria were also clearly described.

## 3. Field Applications of MATS

### 3.1. Application of MATS to Estimate Country-Specific Serogroup B Strain Coverage of 4CMenB

In a large study [5] (1,052 MenB isolates from five countries) carried out in Europe, the results of MATS predicted an overall strain coverage of 78% (95% CI: 63–90%), and half of the tested strains would have been covered by at least two vaccine antigens. The most common MATS phenotypes were fHbp plus NHBA, no antigen, fHbp plus NHBA plus PorA, fHbp, and NHBA; expression of NadA was above the positive bactericidal thresholds in only a small proportion [7% (16/235)] of isolates that harboured the NadA gene. The robustness of estimates was further established by MATS testing of additional MenB isolates from another two countries (Spain and the Czech Republic). Some level of between-country variation in coverage estimates was observed: 87% (95% CI: 70–93%) in Italy, 85% (95% CI: 76–98%) in Norway, 85% (95% CI: 69–93%) in France, 82% (95% CI: 69–92%) in Germany, 74% (95% CI: 58–87%) in the Czech Republic, 73% (95% CI: 57–85%) in England and Wales, and 69% (95% CI: 48–85%) in Spain. However, this difference was not statistically significant at an *α* level of <0.05. A somewhat lower predicted strain coverage of 66% (95% CI: 46–78%) has been reported in Canada. As in the European study, most tested strains could have been targeted by bactericidal antibodies against >1 vaccine antigen. Thus, 29% and 11.5% of isolates were covered by two and three antigens, respectively. The highest relative contribution to coverage was made by fHbp and NHBA [32]. A more recent Greek study has revealed an MATS-predicted coverage of 89.2% (95% CI: 63.5–98.6%) by at least one antigen and 44.5% by at least two antigens. Again, NHBA and fHbp were the greatest contributors to coverage, while PorA and NadA contributed significantly less [33].  Table 2 reports the contribution of each 4CMenB antigen and their combinations to MATS-predicted coverage in three countries.

### 3.2. Application of MATS to Estimate Serogroup X Strain Coverage of 4CMenB

Today, the* N. meningitidis* group X (MenX), which has caused several outbreaks in Africa, remains the only serogroup for which no vaccine exists. The cross serogroup immunogenicity elicited by 4CMenB is biologically plausible, since the majority of capsular strains may express the vaccine antigens [24]. In a paper by Hong et al. [35], nine African MenX isolates and two French MenX isolates were tested by means of MATS in order to determine the presence, diversity, and expression level of 4CMenB antigens. In this study, the PBT values estimated for MenB isolates were applied to MenX strains; the strains were also analyzed by hSBA by using preimmunization and postimmunization sera from different age groups. All African isolates displayed MATS RP values for fHbp above PBT (2.5–5%); conversely, the two French strains showed RP values below the lower limit of quantitation for fHbp, as a result of mismatching for fHbp. Among the African isolates, RPs for NHBA ranged from 11.3% to 31.5%; only three isolates displayed RP values above the PBT. Moreover, one African and one French isolate had the* nadA* gene, but its expression was very low. Although a correlation between MATS RP and hSBA has not yet been established for non-B strains, good agreement was observed between MATS RPs and killing in hSBA in all the age groups for the MenX strains tested in this study.

### 3.3. Application of MATS to Estimate the Potential Impact of 4CMenB on Carriage

The impact of meningococcal vaccination on asymptomatic nasopharyngeal carriage is of primary importance, since healthy carriers are the main reservoir of* N. meningitidis* [36]. Again, MATS may provide useful insights into carriage status, as has been documented in two recent studies [37, 38]. Claus et al. [37] applied MATS to investigate whether 41 capsule-null locus meningococci isolated from Germany, the Czech Republic, and Burkina Faso harbored the genes and expressed the main 4CMenB antigens. However, as PBT had not yet been assessed for capsule-null locus meningococci, PBT values were not applied in their study. Six strains belonging to the clonal complex ST-198 expressed fHbp subvariant 1.4, with RPs ranging from 7.9% to 11.2%. NHBA was expressed in all 41 strains, with RP values of 2.3–58.2%; the highest RPs were seen in isolates belonging to clonal complexes ST-198 and ST-845. The* nadA* gene was found in only one isolate; the expression of the NadA protein was below the limit of detection. No strain matched for the PorA included in 4CMenB. Interestingly, the authors documented that the presence of a capsule has almost no impact on antigen detection levels. In particular, MenB strains MC58 and H44/76 and their corresponding capsule-null isogenic mutants, as well as the capsule-null isolate *α*30 and its encapsulated derivative, showed very similar RPs for fHbp and NHBA [37].

In an Italian longitudinal carrier study [38] 32 out of 173 students tested positive for* N. meningitidis* of different serogroups in at least 1 of 4 examinations. As shown by MATS, strains expressing subvariants of fHbp-1 isolated from 5 students showed detectable expression levels of the antigen, with RPs of 1.2–122.1%. Conversely, isolates carrying fHbp-2 and fHbp-3 were negative on MATS. NHBA was detectable in all isolates (RPs of 2.5–131.2%), while, as expected, NadA expression was detected in only one subject, being NadA very poorly expressed in carrier strains. This study also revealed that RPs for individual antigens remained comparable in subjects on consecutive positive swabs, with the exception of NadA, for which the only positive subject showed a more than 10-fold higher RP on the second swab (43%) than on the first, third, and fourth (3.7%, 3.4%, and 3.1%, resp.).

These two studies [37, 38] have indicated that 4CMenB will very probably affect the carriage and acquisition of* N. meningitidis*; the impact on the unencapsulated strains will probably be less pronounced. It must, however, be borne in mind that, in the case of capsule-null locus strains, the association between hSBA and MATS is quite difficult to establish, owing to their intrinsic susceptibility to complement-mediated killing [38]. In any case, vaccine-induced changes in circulating meningococcal strains and detailed typing of both invasive and carrier isolates should be subject to strict monitoring in the near future.

## 4. MATS May Underestimate 4CMenB Coverage

Intrinsically, MATS is a conservative predictor of MenB strain coverage; indeed, in comparison with hSBA, it underestimates coverage. Frosi et al. [30] applied a stratified proportional random sampling procedure to select a representative panel of 40 MenB isolates from the overall panel of 535 isolates collected in England and Wales over 2007-2008. The selected isolates were tested in the hSBA assay with pooled sera from infant and adolescent vaccinees, and the results compared by means of MATS. In this study, MATS-based predictions of coverage of 70% (95% CI: 55–85%) were largely confirmed by 88% killing in hSBA (95% CI: 72–95%); 27 true positives and 4 true negatives were found, yielding overall accuracy of 78%; positive and negative predictive values were 96% and 33%, respectively [30]. Thus, coverage predicted by MATS has been largely confirmed by subsequent hSBA on pooled infant and adolescent postvaccination sera. This discrepancy between MATS and hSBA assessments of coverage could be explained by various factors.

### 4.1. Non-PorA Components of OMV

One of the four main 4CMenB components is OMV from MenB strain NZ98/254 [39]. OMVs are released by several bacterial species and contain various outer membrane proteins, lipopolysaccharide, and a lumen with periplasmic constituents [40]. Apart from PorA, other proteins identified in the outer membrane have been shown to be immunogenic, including, for example, opacity-associated proteins (Opc and Opa) [41] and* Neisseria* surface protein A (NspA) [42]. MATS ELISA does not detect antibodies elicited against such OMV components.

### 4.2. Synergistic or Additive Effects of Antibodies against Multiple Antigens

MATS does not consider the possible effects of bactericidal antibodies against more than one antigen present in 4CMenB, including different outer membrane proteins. Giuliani et al. [43] have documented that antibodies against non-PorA antigens present in OMV, which may be either bactericidal or not, are able to induce a synergistic bactericidal activity with antibodies against fHbp. Similarly, anti-NHBA antibodies may exert a cooperative activity with antibodies against other antigens [44]. Sera from six subjects immunized with a meningococcal vaccine containing recombinant GNA2091-fHbp, NHBA-GNA1030, and NadA were tested in hSBA both before and after depletion of anti-fHbp and/or anti-NHBA antibodies. All vaccinees showed at least a 4-fold increase in hSBA titers in comparison with their preimmune serum against the test strain H44/76, which matched the fHbp present in the vaccine. When anti-fHbp antibodies were depleted, hSBA titers decreased by at least 88% in all subjects. Four of six vaccinees showed at least a 4-fold increase in hSBA titers against the strain M4407, which expresses a heterologous to the vaccine fHbp-2 and a homologous to the vaccine NHBA amino acid sequence. In one vaccinee, hSBA was directed mostly against NHBA, in another vaccinee against fHbp, while in the remaining two subjects depletion of antibodies against either fHbp or NHBA more than halved the hSBA titer. In all four subjects, depletion of both anti-fHbp and anti-NHBA antibodies suppressed bactericidal activity to a greater degree than depletion of these antibodies individually, indicating a cooperative bactericidal activity between antibodies against fHbp and NHBA. The same study showed that, in mice immunized with only fHbp variant 1 antigen or NHBA vaccine antigen, the percentage survival of strain M4407 incubated with a 1 : 1 mixture of pooled sera from mice immunized with NHBA and pooled sera from mice immunized with fHbp was lower than that when the strain was incubated with a 1 : 1 mixture of pooled sera from mice immunized with NHBA and pooled sera from negative control mice immunized with aluminum hydroxide [44].

### 4.3. NadA* In Vitro* Downregulation

In comparison with both NHBA and fHbp, the relative contribution of NadA to 4CMenB coverage has proved to be very low in all studies conducted so far [5, 14, 32, 33], despite the fact that this antigen is carried by approximately one-third of pathogenic isolates and by three of four hypervirulent lineages. The most probable reason for this observation is the very complex mechanisms of* nadA* gene regulation. The phase-variable expression of nadA is mostly mediated by NadR, which represses* nadA* [45]. Indeed, under* in vitro* conditions in both hSBA and MATS,* nadA* repression by NadR results in inefficient killing of various MenB strains by anti-NadA antibodies. However, it has been suggested that* nadA* expression* in vitro* may differ from that* in vivo*. In this regard, sera from children infected with strains that do not express NadA (RP = 0%) or displaying low NadA expression (RP < PBT) have been seen to recognize NadA recombinant proteins, also confirming the hypothesis that NadA is less expressed* in vitro*; this degree of recognition is much higher than that observed in sera from subjects infected by* nadA* negative strains [46]. Knocking out* nadR* results in a higher level of NadA expression and efficient killing by sera from subjects vaccinated with 4CMenB. Moreover, the MenB strain NGP165, which is mismatched for fHbp, NHBA, and PorA and has NadA RP < PBT, is not killed by sera from infants vaccinated with 4CMenB when it is grown* in vitro*. However, the same infant sera provide passive* in vivo* protection against NGP165 bacteremia, as has been shown in an infant rat model [47].

## 5. Conclusion

This paper attempts to critically review available studies that have used MATS. Implementation of MATS is expected to grow steadily, as 4CMenB has recently been approved for human use in several countries [48–50]. MATS could be useful in tracking spatial and temporal changes in MenB epidemiology and their repercussions on 4CMenB coverage. As anticipated by Vogel et al. [23], there are two main reasons for the increased use of MATS as follows: (i) implementation of the vaccine will potentially affect the population structure of* N. meningitidis*, in that the proportion of strains not covered by 4CMenB will probably increase, thus requiring additional surveillance efforts and (ii) careful assessment of vaccination failures.

A possible limitation of this method could be the lack of information on the relationship between pooled sera, which are used to calculate PBT, and the response rate of individual subjects; this aspect has not yet been fully investigated and is currently under evaluation.

Although MATS has been established for MenB strains, the method is flexible enough to be modified for application to other* N. meningitidis* serogroups and bacteria species. To date, PBT values have been established only for serogroup B, thereby limiting MATS application to this serogroup. However, as the genetic diversity of other serogroups is substantially lower, the establishment of PBTs for these should require a lower number of strains. Moreover, reverse vaccinology is being applied to a number of other bacteria [51], and MATS ELISA could be adapted in order to assess these pathogens [26].

Across the studies conducted so far [5, 32, 33], the MATS-predicted MenB strain coverage provided by 4CMenB has shown some differences among areas or countries, with minimum values observed in Canada and maximum values in Greece. Apart from the diversity in circulating strains, a possible explanation for this variability is that MenB strain panels may not be fully representative, since they generally come from passive surveillance systems [5]. Likewise, it must be taken into account that MATS underestimates the vaccine coverage [30, 41, 46, 47].

Accurate data on the potential strain coverage provided by the new multicomponent vaccine are of crucial interest to policy-makers in order to decide whether to introduce the vaccine into national immunization programs. MenB coverage estimates based on MATS have already been used in various economic evaluations of 4CMenB [52, 53].

In conclusion, MATS is an innovative technique that has several advantages, including (but not limited to) the following:

- assessment of both the level of expression and antigenic relatedness of fHbp, NHBA, and NadA vaccine antigens in meningococci isolates;
- from the technical standpoint, MATS is more rapid and resource-saving than other approaches;
- despite some level of underestimation, MATS strongly correlates with the universally accepted correlate of protection and provides satisfactorily accurate MenB coverage predictions;
- it is highly reproducible and can be successfully transferred to new laboratories;
- it enables near real-time estimation of the postimplementation effectiveness of 4CMenB;
- PBT values may be used to efficiently predict 4CMenB strain coverage on a representative panel of invasive isolates from any geographical setting; such estimates are crucial to economic and health technology assessment (HTA) studies.

## Figures and Tables

**Table 1:** Positive bacterial threshold (PBT) values with 95% CIs for three antigens [26].

Antigen	Estimate %	95% CI
fHbp	2.1	1.4–3.1
NHBA	29.4	16.9–51.1
NadA	0.9	0.4–1.9

**Table 2:** Relative contribution (%) of all possible antigen combinations to MATS-predicted coverage in three countries [5, 30, 32, 33].

Antigen (MATS phenotype)	England and Wales (*n* = 535)	Canada (*n* = 157)	Greece (*n* = 148)
fHbp+/NHBA−/NadA−/PorA−	14.6	12.7	9.5
fHbp−/NHBA+/NadA−/PorA−	7.9	13.4	33.8
fHbp−/NHBA−/NadA+/PorA−	—	—	0.7
fHbp−/NHBA−/NadA−/PorA+	0.4	—	0.7
fHbp+/NHBA+/NadA−/PorA−	29.9	25.5	37.1
fHbp+/NHBA−/NadA+/PorA−	—	0.6	—
fHbp+/NHBA−/NadA−/PorA+	3.2	1.9	—
fHbp−/NHBA+/NadA−/PorA+	0.9	—	1.3
fHbp−/NHBA+/NadA+/PorA−	0.4	0.6	—
fHbp−/NHBA−/NadA+/PorA+	—	—	—
fHbp+/NHBA+/NadA+/PorA−	0.2	—	—
fHbp+/NHBA+/NadA−/PorA+	15.7	11.5	6.1
fHbp+/NHBA−/NadA+/PorA+	—	—	—
fHbp−/NHBA+/NadA+/PorA+	—	—	—
fHbp+/NHBA+/NadA+/PorA+	—	—	—
fHbp−/NHBA−/NadA−/PorA−	26.9	33.8	10.8
